# Frequency and Indications of Non-Musculoskeletal Examinations: A Cross-Sectional Survey of South African Chiropractors

**DOI:** 10.3390/healthcare14131853

**Published:** 2026-06-25

**Authors:** Zanéll Blignaut, Christopher Yelverton

**Affiliations:** Department of Chiropractic, Faculty of Health Sciences, University of Johannesburg, Johannesburg 2094, South Africa; zanellblignaut@icloud.com

**Keywords:** chiropractic, South Africa, primary healthcare, physical examination

## Abstract

**Highlights:**

**What are the main findings?**

- South African chiropractors routinely perform basic vital sign assessments (blood pressure and heart rate) and neurological examinations, but pulmonary, cardiac, abdominal, breast, and genitourinary examinations are conducted infrequently, with the majority of respondents reporting they never perform these assessments.
- Years of clinical experience did not influence examination frequency.

**What are the implications of the main findings?**

- The inconsistent use of complete vital sign sets and low frequency of systemic organ examinations suggest potential gaps in screening for underlying pathologies that may mimic musculoskeletal complaints, highlighting the need for greater standardisation in clinical practice.
- Strengthening continuing professional development programmes in systemic assessment could enhance chiropractors’ capacity to identify red flags, facilitate timely referrals, and support their integration into South Africa’s primary healthcare system.

**Abstract:**

**Background/Objectives:** Chiropractors serve as first-contact practitioners in South Africa and frequently encounter patients with systemic conditions that may mimic musculoskeletal complaints. Non-musculoskeletal (non-MSK) examinations are essential for identifying red flags, ruling out serious pathologies, and facilitating timely referrals. Despite their importance for patient safety and integration into primary healthcare, limited research exists on the frequency with which South African chiropractors perform these assessments. This study aimed to describe the frequency and indications for non-MSK examinations performed by South African chiropractors and to explore variations across examination types, demographic factors, years of experience, and training institutions in secondary analyses. **Methods:** A cross-sectional online survey was distributed to 898 registered chiropractors, yielding 186 responses (20.7%). The questionnaire assessed the frequency of non-MSK examinations using a five-point Likert scale. Data were analysed using descriptive statistics (frequencies, percentages, medians, interquartile ranges). Exploratory subgroup comparisons were conducted using nonparametric tests, but these findings should be interpreted with caution due to small and uneven sample sizes in some subgroups. Ethical approval was obtained (REC-3366-2025). **Results:** Most respondents were female (57.5%) and practising in Gauteng (49.5%). Blood pressure (84.4%) and heart rate (81.2%) were the most frequently performed examinations, while respiratory rate (12.4%), oxygen saturation (9.7%), and temperature (11.8%) were the least frequently performed vital signs. Breast (3.8%), abdominal (10.2%), and genitourinary (1.1%) examinations were rarely conducted. Exploratory subgroup observations suggested provincial variation: chiropractors in KwaZulu-Natal performed non-MSK examinations more frequently than those in Gauteng and the Western Cape (mean differences ranging from 0.21 to 1.19 on a five-point scale), whereas no meaningful differences were found across years in practice. **Conclusions:** South African chiropractors perform a selective range of non-MSK examinations, supporting their role as first-contact practitioners. However, many systemic examinations are conducted infrequently, with observed provincial variation. These descriptive findings highlight the need for greater consistency and standardisation in non-MSK screening to enhance patient safety and interdisciplinary care. Future adequately powered studies are needed to confirm the exploratory subgroup observations.

## 1. Introduction

Chiropractic care focuses on the diagnosis, treatment, and prevention of mechanical conditions of the musculoskeletal (MSK) system and considers the effects on the nervous system and overall health [1,2]. In South Africa, chiropractors are recognised as primary contact practitioners under the Allied Health Professions Council of South Africa (AHPCSA) and are legally authorised to diagnose and manage conditions within their scope of practice [3]. They complete a comprehensive six-year academic and clinical training at either the University of Johannesburg (UJ) or the Durban University of Technology (DUT), which equips them with skills in musculoskeletal assessment, diagnostic imaging, and evidence-based clinical decision-making [4,5]. While traditionally focused on MSK disorders, chiropractors are also trained to identify non-musculoskeletal (non-MSK) conditions to ensure timely referrals and integrated patient care [6].

MSK disorders are among the most common reasons patients seek chiropractic care worldwide and are a major global health burden, contributing significantly to disability and reduced quality of life [7,8]. These conditions, often caused by trauma, repetitive strain, or poor ergonomics, include lower back pain, tendinopathies, carpal tunnel syndrome, and neck pain [8,9]. Chiropractors are frequently consulted for the evaluation and management of these disorders, with clinical guidelines that offer conservative and non-pharmacological interventions. Patients often present with systemic conditions that mimic MSK complaints originating from non-MSK causes [6,10]. For example, cardiac conditions may mimic shoulder or mid-back pain, gastrointestinal conditions may refer pain to the thoracolumbar region, and diabetes mellitus, which can present as peripheral neuropathy resembling lumbar radiculopathy [11]. As first-contact practitioners, chiropractors have a critical responsibility to differentiate between MSK and non-MSK conditions to ensure patient safety through timely referrals and appropriate management [12,13].

In primary healthcare systems, first-contact practitioners are responsible not only for managing presenting complaints but also for screening for underlying conditions that may present atypically. This is particularly critical in low- and middle-income countries such as South Africa, where access to specialist care is limited, and patients may present to any available healthcare provider for initial assessment. Chiropractors, as legally recognised first-contact practitioners in South Africa, therefore occupy a position of significant clinical responsibility. Failure to identify a systemic condition masquerading as a musculoskeletal complaint can result in delayed diagnosis, inappropriate treatment, and adverse patient outcomes.

The ability to perform non-MSK examinations and recognise red flags is essential for chiropractic clinical competence and supports integrated, patient-centred care [14,15]. Non-MSK conditions include disorders of the cardiovascular, respiratory, gastrointestinal, neurological, and endocrine systems [6]. Conditions such as hypertension, chronic obstructive pulmonary disease, epilepsy, and diabetes may present subtly or coexist with MSK complaints [16]. Recognising red flags such as unexplained weight loss, fever, non-mechanical pain, or neurological signs is crucial for patient safety and early intervention [17].

The clinical consequences of missing non-MSK conditions in chiropractic practice are not merely theoretical. Patients with undiagnosed hypertension may be at risk of cerebrovascular events if cervical manipulation is performed without appropriate screening. Undiagnosed diabetes may present with peripheral neuropathy that mimics lumbar radiculopathy, leading to inappropriate spinal manipulation while glycaemic control deteriorates. Similarly, metastatic bone lesions may present as mechanical back pain, and chiropractors must be equipped to recognise red flags that warrant immediate medical referral. These examples underscore that non-MSK examinations are not ancillary to chiropractic care but are fundamental to safe, competent first-contact practice.

Despite this, research on the frequency of non-MSK assessments performed by chiropractors remains limited, especially in the South African context, where challenges to healthcare access highlight the importance of chiropractors in primary care.

The integration of chiropractors into the multidisciplinary healthcare system has attracted increased attention globally. In the South African context, where the burden of undiagnosed chronic conditions such as hypertension, diabetes, and HIV-related disease remains high, the role of all first-contact practitioners, including chiropractors, in early detection and referral is particularly pressing. Research highlights that such collaboration strengthens healthcare delivery, increases communication between practitioners, and improves patient satisfaction [14,18]. In South Africa, chiropractic care largely operates in the private sector, with limited integration into public healthcare and few structured referral systems [19]. Barriers such as poor interprofessional communication, regulatory challenges, and low awareness among other healthcare providers exist. Despite this, chiropractors could play a vital role in reducing the strain on primary care services by treating MSK conditions, screening for non-MSK conditions, and facilitating referrals, particularly in rural and underserved communities [12,20].

A previous study [21] examined non-MSK examinations among chiropractors in Canada, which showed variations in clinical application and highlighted gaps regarding referral patterns and diagnostic outcomes. Given the differences in education, regulation, and healthcare infrastructure in South Africa in comparison to other nations, local research is required. However, there are no comparable data for South Africa, where unique healthcare challenges exist, such as restricted access to primary care and a high prevalence of undiagnosed chronic conditions. This gap in the literature highlights the need to explore chiropractic practice patterns within the South African context.

The aim of this study was to determine the frequency and indications for non-MSK examinations performed by chiropractors in South Africa. By doing so, it examined the extent to which chiropractors use system-based assessments, their readiness to identify non-MSK conditions, and their consideration of their role as primary contact practitioners within an integrated healthcare system. Findings from this research could improve future educational strategies, strengthen interprofessional collaboration, and improve the profession’s contribution to primary healthcare in South Africa.

## 2. Materials and Methods

### 2.1. Study Design

This cross-sectional quantitative study investigated the frequency and indications for non-MSK examinations performed by South African chiropractors. Data were collected using an online questionnaire via QuestionPro (Question, Austin, TX, USA).

The survey was active for 46 days (from 14 April 2025 to 30 May 2025) and was distributed as a survey link via the Allied Health Professions Council of South Africa (AHPCSA) and the Chiropractic Association of South Africa (CASA) databases to registered chiropractors in South Africa.

### 2.2. Population and Eligibility Criteria

Based on personal communication with the Allied Health Professions Council of South Africa, a total of 998 chiropractors were registered with AHPCSA. In their estimation, approximately 100 practitioners may not be working within South Africa without formally updating their registration status [22]. Based on their estimate, the practising population was approximately 898 chiropractors (N = 898), which was used as the target population for this study.

To be eligible, participants had to be registered with the Allied Health Professions Council of South Africa (AHPCSA), work a minimum of 10 clinical hours per week and/or be a lecturer/clinician at the University of Johannesburg or the Durban University of Technology.

Chiropractors who were not primarily practising in one of the nine provinces of South Africa, retired practitioners, or those who no longer practise were excluded.

### 2.3. Sample and Setting

Based on the total population of 898 actively practising chiropractors in South Africa, a minimum sample size of 87 respondents was required to achieve a 95% confidence level with a 10% margin of error, assuming maximum response variability (*p* = 0.5). Sample size was calculated using the standard formula for cross-sectional surveys in finite populations. To account for incomplete responses and ensure robust statistical power, the target sample size was set at 100 respondents.

### 2.4. Instrument Development and Measures

A modified version of the original questionnaire [21] was used as the primary data collection tool. This instrument was adapted to align with the South African chiropractic context while maintaining the integrity of the original content. Specifically, the language was revised, the demographic section was updated to include all nine provinces, and the case study questions were removed, ensuring relevance. The questionnaire was piloted with seven chiropractic staff members (academic and clinical educators) at the University of Johannesburg to evaluate its clarity, relevance, and usability. Participants were asked to complete the survey and provide feedback on question wording, comprehensibility, and overall flow. Minor linguistic refinements were made based on this feedback to enhance clarity. Pilot responses were excluded from the final study sample.

The questionnaire comprised three sections:

Section A: Captured demographic and professional details;
Section B: Assessed the frequency of non-musculoskeletal (non-MSK) examinations using a five-point Likert scale (1 = Never, 2 = At least once/year, 3 = At least once/month, 4 = At least once/week, 5 = At least once/day);
Section C: Explored the reasons why certain examinations were not performed. An electronic information letter and consent form were inserted within the survey, outlining the objectives, the primary researchers’ contact details, and the confidentiality of the data.

### 2.5. Ethical Considerations

The research was approved by the University of Johannesburg Faculty of Health Sciences Research Ethics Committee (REC-3366-2025). Participation was voluntary and anonymous. This study aligns with the profession’s best interests by examining South African chiropractors’ current practices regarding non-MSK examinations. This will help clarify their role as primary-contact practitioners and guide future educational and clinical approaches to improving patient care.

All participants were required to read both the information and the consent form, which outlined the purpose, the researcher’s details, and potential benefits. Consent was obtained electronically by selecting “Agree and continue with survey.” To access the online survey, participants had to provide consent. No identifying data were collected during data collection, and all participants remained anonymous.

During the preparation of this work, the authors used DeepSeek (DeepSeek-V3, DeepSeek AI) to improve the readability and language of the manuscript. After using this tool, the authors reviewed and edited the content as needed and take full responsibility for the final content of the publication. No AI tools were used to generate data, perform analyses, or draw scientific conclusions.

### 2.6. Data Analysis

The data were collected and processed using the IBM SPSS Statistics (version 30) for statistical analysis, with assistance from Statistical Consultation Services (STATKON) at the University of Johannesburg. No missing data were reported. Frequencies and percentages were used to characterise categorical data. Descriptive statistics (frequencies, percentages, medians, and interquartile ranges) were used to summarise the data.

For descriptive statistics, the Kolmogorov–Smirnov and Shapiro–Wilk normality tests were conducted. It was established that some of the total and component domains were not normally distributed, and non-parametric statistical tests (Mann–Whitney U and Kruskal–Wallis) were used for the analysis.

## 3. Results

### 3.1. Response Rate

A valid response rate of 20.7% (*n* = 186) was achieved from a total population of 898 AHPCSA-registered chiropractors practicing in South Africa. This exceeded the target sample size of 100 respondents.

### 3.2. Demographic Data

The majority of respondents were female (*n* = 107; 57.5%), followed by male (*n* = 77; 41.4%) and other/prefer not to say (*n* = 2; 1.1%). In terms of years in practice, the largest proportion had 0–5 years of experience (*n* = 69; 37.1%), followed by 5–10 years (*n* = 41; 22.0%), 21+ years (*n* = 39; 21.0%), 11–15 years (*n* = 22; 11.8%), and 16–20 years (*n* = 15; 8.1%). The majority of respondents worked 21–40 h per week (*n* = 85; 45.7%) or 41–60 h per week (*n* = 60; 32.3%). Geographically, participants were distributed across eight of the nine provinces, with the highest representation from Gauteng (*n* = 92; 49.5%), followed by KwaZulu-Natal (*n* = 40; 21.5%) and the Western Cape (*n* = 35; 18.8%). A full summary of demographic data is presented in Table 1.

### 3.3. Frequency of Non-MSK Examination

For all frequency tables, responses were collapsed into three categories: Never, Infrequent (at most once per year or once per month as indicated), and Regular (at least once per week). Medians and interquartile ranges (IQR) are reported as measures of central tendency and dispersion, which are more appropriate than means for ordinal Likert-type data.

#### 3.3.1. Physical Examination

**Figure 1:**
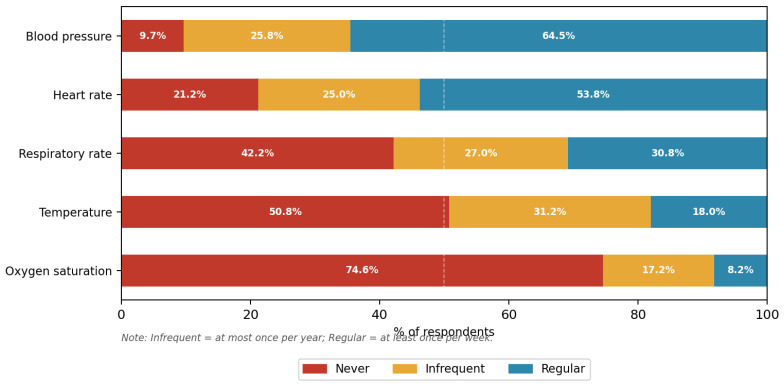
Frequency of physical (vital signs) examination (*n* = 186).

Blood pressure and heart rate were the most frequently performed vital signs. Nearly half of respondents (*n* = 90; 48.4%) reported measuring blood pressure daily, and 40.8% (*n* = 75) assessed heart rate with a similar regularity. In contrast, oxygen saturation and temperature were rarely assessed, with 74.6% (*n* = 138) and 50.8% (*n* = 93) of respondents, respectively, reporting never performing these tests (Figure 1).

#### 3.3.2. Neurological Examination

**Figure 2:**
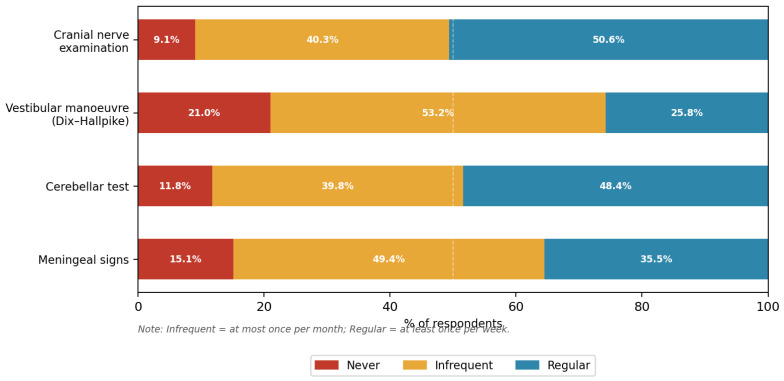
Frequency of neurological examinations (N = 186).

Neurological examinations were performed relatively frequently. Cranial nerve examination was the most commonly performed assessment, with 27.4% (*n* = 51) of respondents conducting it at least once daily. Cerebellar tests were also regularly included in practice, typically performed at least weekly. Vestibular manoeuvres (e.g., Dix–Hallpike test) were performed less frequently (Figure 2).

#### 3.3.3. Infrequently Performed Examinations

##### Ophthalmic Examination

Observation of the fundus was never performed by 65.1% (*n* = 121) of respondents. Only 13.0% (*n* = 24) performed this examination regularly (at least once per week), with a median frequency of 1.0 (IQR: 1–1).

##### Otorhinolaryngology Examination

Observation of the eardrum was never performed by 60.8% (*n* = 113) of respondents. Only 6.5% (*n* = 12) performed this examination regularly, with a median frequency of 1.0 (IQR: 1–1).

##### Pulmonary Examination

Pulmonary auscultation and percussion were never performed by 48.1% (*n* = 89) of respondents. Only 10.2% (*n* = 19) performed this examination regularly, with a median frequency of 2.0 (IQR: 1–2).

##### Cardiac Examination

Cardiac auscultation and percussion were never performed by 51.6% (*n* = 96) of respondents. Only 9.1% (*n* = 17) performed this examination regularly, with a median frequency of 1.0 (IQR: 1–2).

#### 3.3.4. Peripheral Vascular Examination

**Figure 3:**
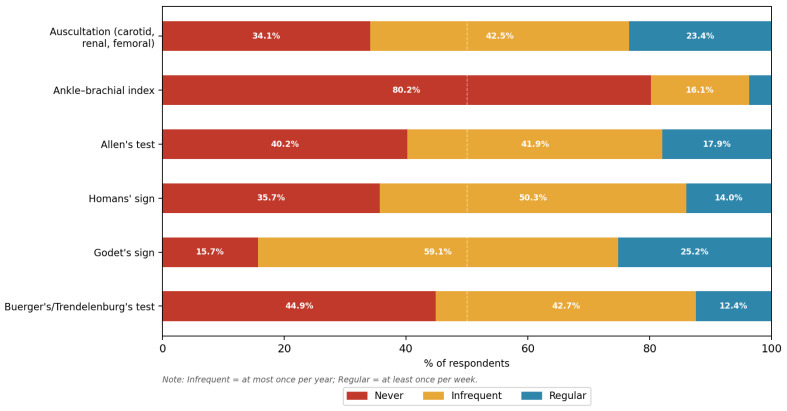
Frequency of peripheral vascular examination (N = 186).

Auscultation of the carotid arteries, abdominal aorta, renal arteries, and femoral arteries was never performed by 34.1% (*n* = 63) of respondents, while 23.4% (*n* = 43) performed it regularly. The ankle–brachial index was the least frequently performed vascular assessment, with 80.2% (*n* = 146) reporting that they never performed it (Figure 3).

#### 3.3.5. Abdominal Examination

**Figure 4:**
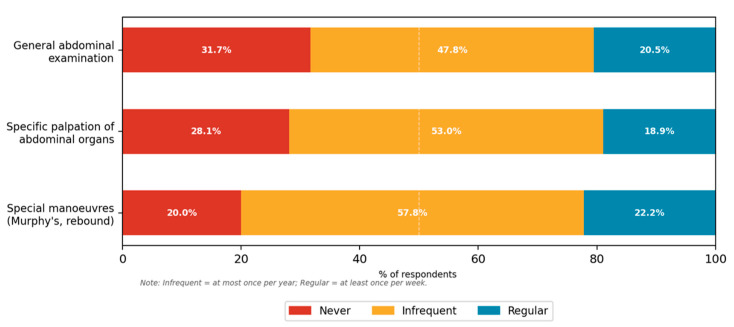
Frequency of abdominal examination (N = 186).

General abdominal examination was never performed by 31.7% (*n* = 59) of respondents. Special manoeuvres (e.g., Murphy’s sign, rebound tenderness) were more frequently conducted, with 22.2% (*n* = 41) performing them regularly (Figure 4).

#### 3.3.6. Breast Examination

**Figure 5:**
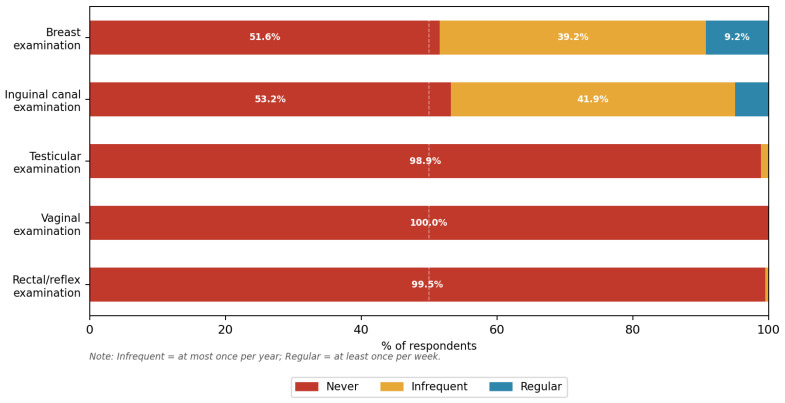
Frequency of breast and genitourinary examination (N = 186).

Breast examination was rarely performed, with 51.6% (*n* = 96) of respondents indicating that they had never conducted this assessment. Only 9.2% (*n* = 17) reported performing it regularly (Figure 5).

#### 3.3.7. Genitourinary Examination

The majority of respondents reported never performing genitourinary examinations. Vaginal examination was not performed by any respondent (100%, *n* = 186). Rectal examination and testicular examination were almost never conducted (99.5% and 98.9% never, respectively). Inguinal canal examination was the most frequently performed in this category, although still limited, with 53.2% (*n* = 99) reporting “never” and 4.9% (*n* = 9) reporting regular performance (Figure 5).

### 3.4. Patient Encounters and Clinical Decision-Making

Of the 186 respondents, 175 provided valid responses, which estimated the percentage of new patients who required a non-MSK examination. The mean percentage reported was 15.96% (SD = 23.65), with a median of 10%, indicating that chiropractors encounter non-MSK cases relatively infrequently (Table 2).

Among new patients requiring a non-MSK examination, the average percentage of cases in which chiropractors reported performing such an examination was 58.84% (SD = 41.57), with a median of 80%. This suggests that while relatively few patients present with non-MSK conditions, examinations are performed in most relevant cases.

**Figure 6:**
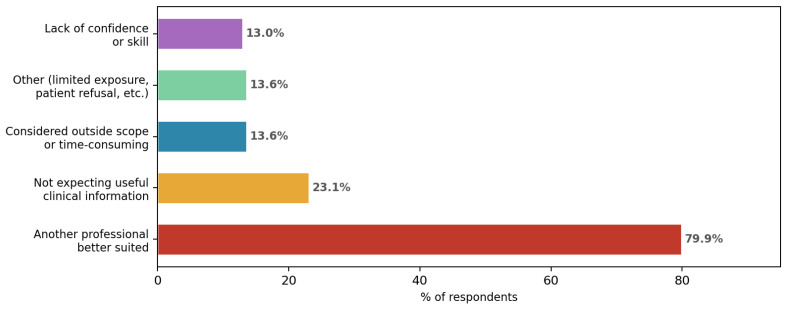
Reasons for not performing a non-MSK examination (N = 186; multiple responses permitted).

In cases where chiropractors did not perform a non-MSK examination on patients who would have required one, the most frequently reported reason was that another healthcare professional would be better suited to perform or interpret the examination (79.9%). Other reasons included not expecting to gain useful clinical information (23.1%), lack of confidence or skill (13.0%), and perceiving such examinations as outside their professional scope or too time-consuming (13.6%). These findings are summarised in Figure 6.

### 3.5. Exploratory Subgroup Analysis

The following subgroup analyses are presented solely for exploratory descriptive purposes. Due to small and uneven sample sizes in some provinces, and the number of comparisons performed, these findings should be interpreted with caution and considered hypothesis-generating rather than confirmatory.

#### 3.5.1. Comparison by Institution

Table 3 presents descriptive comparisons of non-MSK examination frequencies between graduates of the University of Johannesburg (UJ) and the Durban University of Technology (DUT). Mean scores (with 95% confidence intervals) are presented for descriptive purposes.

For most examination domains, mean scores were similar between UJ and DUT graduates, including physical, neurological, peripheral vascular, breast, rectal, vaginal, testicular, and inguinal examinations.

Small differences in mean scores (all less than 0.4 on the five-point scale) were observed for five domains: ophthalmic (UJ: 1.53, DUT: 1.72), otorhinolaryngology (UJ: 1.61, DUT: 1.93), pulmonary (UJ: 2.12, DUT: 2.46), cardiac (UJ: 2.06, DUT: 2.43), and abdominal examinations (UJ: 2.34, DUT: 2.63). In each instance, DUT graduates reported slightly higher frequencies of performing these examinations. Given the modest magnitude of these differences (all < 0.4), the overall examination practices of UJ and DUT graduates appear broadly similar.

#### 3.5.2. Comparison by Years in Practice

Table 4 presents descriptive comparisons of non-MSK examination frequencies between chiropractors with ≤10 years of experience and those with >10 years of experience. Mean scores were similar between the two groups across all examination domains, with differences generally less than 0.2 on the five-point scale. The largest observed difference was for cardiac examinations (≤10 years: 1.85, >10 years: 1.51; difference: 0.34). These findings suggest that years of clinical experience are not associated with meaningful differences in the frequency of non-MSK examinations.

#### 3.5.3. Comparison by Province

Sample sizes for provinces other than Gauteng (*n* = 92), KwaZulu-Natal (*n* = 40), and Western Cape (*n* = 35) were very small (1–6 respondents). Analyses are therefore limited to these three provinces.

Table 5 presents descriptive comparisons of non-MSK examination frequencies across the three major provinces: Gauteng (GP), Western Cape (WC), and KwaZulu-Natal (KZN).

#### 3.5.4. Observed Differences Between Provinces

For seven examination domains, mean scores for KwaZulu-Natal were higher than those for Gauteng and Western Cape (Table 6).

The largest observed differences were for cardiac (0.98–1.19) and pulmonary examinations (0.76–0.94). Gauteng and Western Cape demonstrated similar mean scores across all domains, with no notable differences.

### 3.6. Summary of Descriptive Findings

This descriptive survey found that:

- Most frequently performed examinations: Blood pressure, heart rate, and cranial nerve examinations were performed regularly by the majority of respondents.
- Least frequently performed examinations: Genitourinary examinations were almost never performed (99–100% never). Pulmonary, cardiac, and breast examinations were never performed by approximately half of the respondents.
- Patient encounters: Respondents estimated that 16% of new patients require a non-MSK examination, and such examinations are performed in approximately 59% of indicated cases.
- Reasons for non-performance: The most common reason cited for not performing a non-MSK examination was that another healthcare professional would be better suited (79.9%).
- Subgroup observations (exploratory): Small descriptive differences were observed between institutions and provinces, with KwaZulu-Natal showing higher mean scores for several examination domains. Years of experience showed little association with examination frequency. These exploratory findings should be interpreted with caution due to small and uneven sample sizes in some subgroups.

## 4. Discussion

This descriptive study provides the first overview of non-musculoskeletal (non-MSK) examination practices among South African chiropractors. The findings reveal that while South African chiropractors regularly perform certain non-MSK assessments, particularly vital signs (blood pressure and heart rate) and neurological examinations, many systemic examinations are conducted infrequently. Notably, genitourinary, breast, and most systemic organ examinations (pulmonary, cardiac, abdominal) were rarely performed, with the majority of respondents reporting that they never conducted these assessments.

Exploratory subgroup analyses suggested provincial variations, with chiropractors in KwaZulu-Natal consistently reporting higher frequencies of performing non-MSK examinations than those in Gauteng and the Western Cape. However, these observed differences were modest in magnitude, with mean differences generally below 0.6 on the five-point scale, and should be interpreted with caution due to small and uneven sample sizes in some provinces. Years of clinical experience did not substantially influence examination frequency, suggesting consistent practice patterns across career stages. Similarly, while some observed differences were found between graduates of the two South African training institutions (UJ and DUT), the absolute differences were small (<0.4 on the five-point scale) and unlikely to be clinically meaningful.

The findings are broadly consistent with international research on chiropractic practice patterns. Giroux et al. (2024) [21] reported similar findings in a Quebec-based survey, noting that blood pressure and heart rate were the most frequently performed non-MSK examinations, whereas genitourinary and breast examinations were rarely performed. The consistency across these studies suggests that chiropractors worldwide may share similar perceptions about which non-MSK assessments fall within their scope of practice.

The low frequency of performing complete vital sign sets (particularly respiratory rate and temperature) observed in both studies raises important clinical considerations. In primary care settings, a full set of vital signs is considered essential when evaluating patients presenting with chest pain, headache, or suspected infection [17]. Limited assessment of respiratory rate or temperature could delay recognition of urgent conditions such as hypertension, respiratory distress, systemic infection, or sepsis. This pattern may reflect chiropractors’ primary focus on musculoskeletal presentations, but it also highlights an area where practice could be strengthened to better align with primary care standards.

Goncalves et al. (2021) [6] emphasised the importance of chiropractors recognising non-MSK conditions, given their role as first-contact practitioners. The present findings suggest that while South African chiropractors demonstrate readiness to identify certain non-MSK presentations (particularly through neurological screening), there remains considerable variability in the application of systemic assessments. This variability mirrors broader international trends and underscores the need for context-specific guidance.

The high frequency of blood pressure and heart rate measurement (48.4% and 40.8% daily, respectively) is encouraging and suggests that South African chiropractors recognise the importance of basic cardiovascular screening. Similarly, the regular performance of cranial nerve and cerebellar examinations (median frequencies of 4.0 and 3.0, respectively) indicates that neurological assessment is well integrated into routine chiropractic practice. This supports the profession’s role as first-contact practitioners, equipped to identify potential red flags that require medical referral.

The relatively frequent use of special abdominal manoeuvres (e.g., Murphy’s sign, rebound tenderness) compared to general abdominal examination is noteworthy. This may reflect a targeted approach where chiropractors perform specific tests based on clinical presentation rather than routine systematic screening. While clinically appropriate, this pattern may also indicate that non-MSK examinations are primarily triggered by specific symptoms rather than incorporated as standard practice.

The very low frequency of genitourinary examinations (99–100% never performed) was expected and appropriate given the scope of chiropractic practice in South Africa. As no internal consistency analysis was performed for this domain (given the near-universal “never” responses), these findings simply reflect that these procedures fall outside routine chiropractic practice. This aligns with regulatory frameworks and educational curricula that do not include such assessments within the chiropractic scope [3].

More concerning is the infrequent performance of pulmonary (48.1% never), cardiac (51.6% never), and abdominal examinations (31.7% never for general examination). Respiratory conditions such as chronic obstructive pulmonary disease and asthma may present with referred pain to the shoulder or thoracic spine [16]. Similarly, cardiac ischaemia can manifest as left arm or shoulder pain, and gastrointestinal conditions may refer pain to the thoracolumbar region [11]. Without adequate systemic screening, chiropractors risk missing underlying pathologies that mimic musculoskeletal complaints, potentially delaying appropriate medical care.

The low frequency of ophthalmic (65.1% never) and otorhinolaryngology examinations (60.8% never) is less concerning, as these assessments are less relevant to typical chiropractic presentations. However, certain neurological conditions (e.g., intracranial hypertension, vestibular disorders) may present with visual or otological symptoms that warrant examination [6].

Chiropractors in this study estimated that approximately 16% of new patients require a non-MSK examination, and that such examinations are performed in approximately 59% of these cases. This suggests that while non-MSK presentations are relatively uncommon in chiropractic practice, most practitioners perform appropriate assessments when indicated. However, the wide standard deviations (23.65 and 41.57, respectively) indicate considerable variability in how chiropractors perceive and respond to non-MSK presentations.

The most frequently cited reason for not performing a non-MSK examination was the belief that another healthcare professional is better suited to perform or interpret the results (79.9%). This reflects a collaborative rather than avoidance-based approach and demonstrates an understanding of interprofessional boundaries and referral responsibilities. However, 13.0% of respondents indicated a lack of confidence or skill, and 13.6% perceived such examinations as outside their scope or too time-consuming. These findings suggest that while most chiropractors recognise when referral is appropriate, some may benefit from additional training or clearer scope-of-practice guidelines.

The exploratory observation that chiropractors in KwaZulu-Natal reported higher frequencies of non-MSK examinations compared to those in Gauteng and the Western Cape warrants careful interpretation. Given the exploratory nature of these analyses and the modest magnitude of observed differences (mean differences generally below 0.6 on the five-point scale), these findings should be considered hypothesis-generating rather than confirmatory. The largest observed differences were in cardiac (0.98–1.19) and pulmonary examinations (0.76–0.94), suggesting possible variation in cardiopulmonary screening practices that warrants further investigation.

Several hypotheses may explain these provincial differences:

- Undergraduate Training: Although chiropractic curricula in South Africa are nationally standardised, differences in teaching emphasis or clinical supervision styles between institutions may contribute to subtle practice variations. The Durban University of Technology (DUT) is located in KwaZulu-Natal, and its graduates may be overrepresented in this province. However, the descriptive analysis revealed only small differences between DUT and UJ graduates overall, suggesting that training alone is unlikely to explain the provincial variation observed.
- Patient Demographics: Regional differences in the prevalence of chronic conditions may influence clinical practice. KwaZulu-Natal has a high burden of HIV, tuberculosis, and cardiovascular disease [23]. Chiropractors practising in regions with higher disease prevalence may encounter more patients with systemic presentations, potentially reinforcing the importance of non-MSK screening. This hypothesis is supported by the observation that the largest provincial differences were observed for cardiac and pulmonary examinations, systems commonly affected by conditions prevalent in KwaZulu-Natal.
- Healthcare Integration: The degree of integration with other healthcare professionals may influence examination patterns. Chiropractors working within stronger referral networks or closer collaboration with medical practitioners may be more proactive in identifying and referring patients with potential non-MSK conditions. However, this hypothesis requires further investigation.

Importantly, Gauteng and the Western Cape demonstrated comparable practices with no significant differences between them. This suggests that the observed variation is specific to KwaZulu-Natal rather than reflecting a broader pattern of provincial differences. Understanding why chiropractors in KwaZulu-Natal report higher examination frequencies remains a question for future research.

The descriptive findings highlight both strengths and areas for improvement in South African chiropractic practice. On the one hand, the frequent use of neurological examinations and basic vital signs reinforces the profession’s capacity to function as first-contact practitioners and identify potential red flags. On the other hand, the inconsistent use of complete vital sign sets and low frequency of pulmonary, cardiac, and abdominal examinations suggest that some patients with underlying systemic conditions may be missed. Strengthening the consistency of non-MSK screening could enhance early detection of conditions such as hypertension, undiagnosed diabetes, or respiratory disease, facilitating timely referral and improving patient outcomes.

The finding that 13.0% of respondents lacked confidence in performing non-MSK examinations is concerning and suggests that some practitioners may benefit from additional training or clinical support. This is particularly important given that chiropractors function as primary contact practitioners and may be the first healthcare professional to encounter patients with undiagnosed systemic conditions.

The observation that years of experience did not influence examination frequency suggests that practice patterns are established early in careers and may persist without intervention. This highlights the importance of ensuring that new graduates are adequately prepared to recognise and respond to non-MSK presentations. Continuing professional development programmes should address identified gaps, particularly in pulmonary and cardiac assessments.

The low frequency of certain examinations (e.g., respiratory rate, temperature) may reflect assumptions about what constitutes “routine” chiropractic care. Continuing professional development programmes should explicitly address the importance of complete vital sign assessments in appropriate clinical contexts, emphasising patient safety and the chiropractor’s role in early detection of systemic disease.

The finding that most chiropractors refer patients when uncertain (79.9% citing another professional as better suited) reflects appropriate recognition of professional boundaries. However, effective referral requires the ability to recognise when referral is necessary. Strengthening non-MSK examination skills may enhance chiropractors’ capacity to make appropriate referral decisions, ensuring that patients receive timely care from the most suitable provider.

Greater integration of chiropractors into multidisciplinary teams could facilitate shared care and improve patient outcomes. In South Africa, where healthcare resources are strained and access to primary care is limited in rural areas, chiropractors could play a valuable role in screening for common chronic conditions and facilitating referrals [12,20]. However, realising this potential requires consistent clinical standards, clear scope-of-practice guidelines, and effective communication channels with medical colleagues.

### 4.1. Strengths

This study has several strengths. It is the first investigation of non-MSK examination practices among South African chiropractors, addressing an important gap in the literature. The sample size (*n* = 186) exceeded the minimum required for statistical power (*n* = 87) and is comparable to similar international surveys [21]. The use of a descriptive instrument adapted from previous research enhances comparability with international findings. The inclusion of multiple descriptive comparison groups (institution, years of experience, province) provides a comprehensive description of factors that may be associated with clinical practice.

### 4.2. Limitations

Several limitations should be considered when interpreting these findings. First, the 20.7% response rate raises the possibility of non-response bias. Chiropractors with a particular interest in non-MSK examinations may have been more likely to participate, potentially leading to an overestimation of the frequency of these assessments. Conversely, chiropractors who never perform such examinations may have been less likely to respond. Without data on non-respondents, the direction and magnitude of this bias cannot be quantified. Readers should therefore interpret the reported frequencies as representing the practices of participating chiropractors rather than necessarily generalising to the entire population of South African chiropractors.

Second, the cross-sectional, self-reported design introduces potential for recall and social desirability bias. Participants may have over-reported examinations that they believe they “should” perform or under-reported those that they perceive as outside their scope. Without objective verification (e.g., chart reviews, direct observation), the accuracy of self-reported frequencies cannot be confirmed.

Third, the use of convenience sampling through professional databases (AHPCSA and CASA) represents an additional limitation. Chiropractors who are members of these organisations may differ systematically from non-members in their practice patterns, attitudes, or professional engagement. Furthermore, the voluntary nature of survey participation may have introduced self-selection bias.

Fourth, the uneven distribution of respondents across provinces limits generalisability, particularly for regions with small sample sizes. While the three major provinces (Gauteng, KwaZulu-Natal, Western Cape) were adequately represented, other provinces had very few respondents (1–6 each), and no responses were received from the Northern Cape. The findings cannot be reliably extrapolated to these underrepresented regions. Consequently, the exploratory provincial comparisons should be interpreted with particular caution.

Fifth, the large number of descriptive comparisons presented increases the risk of chance findings. No statistical adjustments were applied, as the subgroup analyses are presented as exploratory descriptive observations rather than confirmatory hypothesis tests. Readers should therefore avoid overinterpreting small differences between groups.

Sixth, the questionnaire was adapted from a Canadian instrument. Differences in training, regulation, and healthcare infrastructure between Canada and South Africa may limit the instrument’s applicability, although the adaptation and piloting process sought to address these.

Finally, the study did not collect data on the reasons for provincial variation, limiting its ability to explain the observed differences. Factors such as patient demographics, local disease prevalence, referral networks, and participation in continuing education remain speculative hypotheses requiring further investigation.

### 4.3. Future Research Directions

This study identifies several avenues for future research. Qualitative methods (e.g., interviews, focus groups) could provide a deeper understanding of the contextual factors underlying provincial variation and clinical decision-making regarding non-MSK examinations. Exploring chiropractors’ experiences, attitudes, and perceived barriers to systemic screening would complement the descriptive findings.

Observational studies using chart reviews or clinical observations could validate self-reported examination frequencies and provide more accurate estimates of actual practice patterns. Comparing self-reported data with clinical records would help quantify the extent of social desirability and recall bias.

Research examining patient outcomes associated with non-MSK screening practices would clarify the clinical impact of these assessments. Such studies could investigate whether chiropractors who perform more frequent systemic screening detect more undiagnosed conditions, refer patients earlier, or achieve better health outcomes. Answering these questions would strengthen the evidence base for chiropractic’s role in primary care.

Longitudinal studies could examine whether practice patterns change over time in response to continuing education, regulatory changes, or increased integration with other healthcare providers. Understanding how clinical behaviours evolve could inform strategies for promoting evidence-based practice.

Finally, adequately powered studies with larger and more representative samples—particularly from underrepresented provinces—are needed to confirm or refute the exploratory provincial differences observed in this study.

## 5. Conclusions

This study provides the first descriptive overview of non-MSK examination practices among South African chiropractors. The findings demonstrate that while chiropractors regularly perform certain assessments (blood pressure, heart rate, neurological examinations), many systemic examinations are conducted infrequently. Years of clinical experience did not show meaningful differences in practice patterns, suggesting consistent habits across career stages.

The low frequency of pulmonary, cardiac, and abdominal examinations raises concerns about potential missed diagnoses, particularly given chiropractors’ role as primary contact practitioners. However, the very low frequency of genitourinary examinations reflects appropriate recognition of professional boundaries. Most chiropractors refer patients when they are uncertain, demonstrating awareness of their interprofessional responsibilities.

The descriptive findings highlight the need for greater consistency and standardisation in non-MSK screening within South African chiropractic practice. Strengthening undergraduate curricula, expanding continuing professional development opportunities, and developing clear clinical guidelines could enhance chiropractors’ competence and confidence in systemic assessment. Such improvements would support the profession’s integration into primary healthcare and ultimately benefit patient safety and outcomes.

Future research should explore the reasons underlying provincial variation, validate self-reported practices through observational methods, and examine the impact of non-MSK screening on patient outcomes. Adequately powered studies with representative samples are needed to confirm the exploratory observations reported here. By addressing these questions, the chiropractic profession can continue to evolve as a valued contributor to South Africa’s healthcare system.

## Figures and Tables

**Table 1:** Summary of demographic data of participants.

Category	Variable	Number of Respondents N (%)
Gender	Male	77 (41.4)
	Female	107 (57.5)
	Other/Prefer not to say	2 (1.1)
Hours in practice	≤10	6 (3.2)
	11–20	31 (16.7)
	21–40	85 (45.7)
	41–60	60 (32.3)
	61–80	4 (2.2)
Years in practice	Less than 5	69 (37.1)
	5–10	41 (22.0)
	11–15	22 (11.8)
	16–20	15 (8.1)
	21+	39 (21)
Employed in a South African teaching institution	No	172 (92.5)
	Yes, full time lecturer	1 (0.5)
	Yes, part-time lecturer	3 (1.6)
	Yes, part time clinician	11 (5.9)
Provinces	Gauteng	92 (49.5)
	Western Cape	35 (18.8)
	KwaZulu-Natal	40 (21.5)
	Eastern Cape	3 (1.6)
	Free State	3 (1.6)
	Limpopo	2 (1.1)
	Mpumalanga	6 (3.2)
	North West	1 (0.5)
Qualification Institution	University of Johannesburg (UJ)	96 (51.6)
	Durban University of Technology (DUT)	57 (30.6)
	Technikon Witwatersrand	12 (6.5)
	Technikon Natal	12 (6.5)
	Other: - Life University - Palmer College - Palmer University - University of Western States - Northwestern College of Chiropractic	9 (4.8)

**Table 2:** Estimated patient encounters requiring non-MSK examinations (N = 175).

Variable	*n*	Mean (%)	SD	Median (%)
Estimated percentage of new patients requiring a non-MSK examination	175	15.96	23.65	10
Percentage of relevant cases where a non-MSK examination was performed	175	58.84	41.57	80

**Table 3:** Comparison of non-MSK examinations between institutions (UJ vs. DUT).

Examination	Institution	Mean	95% CI
Physical	UJ	2.56	2.36–2.76
	DUT	2.85	2.59–3.10
Neurological	UJ	3.03	2.85–3.21
	DUT	3.16	2.91–3.40
Ophthalmic	UJ	1.53	1.34–1.72
	DUT	1.72	1.49–1.96
Otorhinolaryngology (ENT)	UJ	1.47	1.31–1.63
	DUT	1.90	1.64–2.16
Pulmonary	UJ	1.75	1.55–1.94
	DUT	2.17	1.89–2.46
Cardiac	UJ	1.65	1.46–1.83
	DUT	2.13	1.85–2.42
Peripheral vascular	UJ	2.08	1.93–2.23
	DUT	2.29	2.07–2.50
Abdominal	UJ	2.34	2.13–2.54
	DUT	2.63	2.41–2.85
Breast	UJ	1.17	1.08–1.25
	DUT	1.25	1.13–1.36
Rectal reflex	UJ	1.00	1.00–1.00
	DUT	1.01	0.99–1.04
Vaginal	UJ	1.00	1.00–1.00
	DUT	1.00	1.00–1.00
Testicular	UJ	1.01	0.99–1.03
	DUT	1.01	0.99–1.04
Inguinal	UJ	1.60	1.43–1.77
	DUT	1.81	1.58–2.05

**Table 4:** Comparison of non-MSK examinations by years in practice (≤10 vs. >10 years).

Examination	Years in Practice	Mean	95% CI
Physical	≤10 years	2.74	2.54–2.93
	>10 years	2.48	2.35–2.87
Neurological	≤10 years	3.10	2.93–3.27
	>10 years	2.97	2.81–3.32
Ophthalmic	≤10 years	1.66	1.48–1.85
	>10 years	1.54	1.29–1.76
Otorhinolaryngology (ENT)	≤10 years	1.63	1.47–1.79
	>10 years	1.49	1.44–1.95
Pulmonary	≤10 years	1.88	1.69–2.07
	>10 years	1.66	1.73–2.32
Cardiac	≤10 years	1.85	1.66–2.05
	>10 years	1.51	1.60–2.17
Peripheral vascular	≤10 years	2.10	1.95–2.24
	>10 years	2.08	2.05–2.46
Abdominal	≤10 years	2.38	2.22–2.55
	>10 years	2.19	2.28–2.83
Breast	≤10 years	1.18	1.10–1.26
	>10 years	1.09	1.11–1.33
Rectal reflex	≤10 years	1.00	1.00–1.00
	>10 years	1.00	0.99–1.04
Vaginal	≤10 years	1.00	1.00–1.00
	>10 years	1.00	1.00–1.00
Testicular	≤10 years	1.01	1.00–1.00
	>10 years	1.00	0.99–1.06
Inguinal	≤10 years	1.62	1.45–1.79
	>10 years	1.46	1.60–2.03

**Table 5:** Comparison of non-MSK examinations by province (GP, WC, KZN).

Examination	Province	Mean	95% CI
Physical	GP	2.63	2.42–2.85
	WC	2.48	2.15–2.82
	KZN	3.04	2.70–3.38
Neurological	GP	3.04	2.85–3.24
	WC	2.97	2.65–3.29
	KZN	3.28	2.93–3.62
Ophthalmic	GP	1.57	1.36–1.77
	WC	1.54	1.17–1.92
	KZN	1.80	1.47–2.13
Otorhinolaryngology (ENT)	GP	1.60	1.40–1.80
	WC	1.49	1.17–1.80
	KZN	1.95	1.63–2.27
Pulmonary	GP	1.84	1.61–2.06
	WC	1.66	1.35–1.97
	KZN	2.60	2.18–3.02
Cardiac	GP	1.72	1.50–1.93
	WC	1.51	1.25–1.78
	KZN	2.70	2.28–3.12
Peripheral vascular	GP	2.09	1.93–2.24
	WC	2.08	1.84–2.32
	KZN	2.46	2.12–2.80
Abdominal	GP	2.42	2.20–2.64
	WC	2.19	1.92–2.45
	KZN	2.79	2.47–3.12
Breast	GP	1.17	1.08–1.26
	WC	1.09	0.99–1.18
	KZN	1.38	1.20–1.55
Rectal reflex	GP	1.01	0.99–1.03
	WC	1.00	1.00–1.00
	KZN	1.00	1.00–1.00
Vaginal	GP	1.00	1.00–1.00
	WC	1.00	1.00–1.00
	KZN	1.00	1.00–1.00
Testicular	GP	1.01	0.99–1.03
	WC	1.00	1.00–1.00
	KZN	1.00	1.00–1.00
Inguinal	GP	1.66	1.48–1.84
	WC	1.46	1.20–1.71
	KZN	2.03	1.70–2.35

**Table 6:** Mean scores per region demonstrating observed differences.

Examination	GP Mean	WC Mean	KZN Mean	Observed Difference (KZN-GP)	Observed Difference (KZN-WC)
Physical	2.63	2.48	3.04	0.41	0.56
Otorhinolaryngology	1.60	1.49	1.95	0.35	0.46
Pulmonary	1.84	1.66	2.60	0.76	0.94
Cardiac	1.72	1.51	2.70	0.98	1.19
Abdominal	2.42	2.19	2.79	0.37	0.60
Breast	1.17	1.09	1.38	0.21	0.29
Inguinal	1.66	1.46	2.03	0.37	0.57

## Data Availability

The raw data supporting the conclusions of this article will be made available by the authors on request, but access is restricted to protect participant privacy and confidentiality.
